# A database and API for variation, dense genotyping and resequencing data

**DOI:** 10.1186/1471-2105-11-238

**Published:** 2010-05-11

**Authors:** Daniel Rios, William M McLaren, Yuan Chen, Ewan Birney, Arne Stabenau, Paul Flicek, Fiona Cunningham

**Affiliations:** 1 European Bioinformatics Institute, Wellcome Trust Genome Campus, Hinxton, Cambridge, CB10 1SD, UK

## Abstract

**Background:**

Advances in sequencing and genotyping technologies are leading to the widespread availability of multi-species variation data, dense genotype data and large-scale resequencing projects. The 1000 Genomes Project and similar efforts in other species are challenging the methods previously used for storage and manipulation of such data necessitating the redesign of existing genome-wide bioinformatics resources.

**Results:**

Ensembl has created a database and software library to support data storage, analysis and access to the existing and emerging variation data from large mammalian and vertebrate genomes. These tools scale to thousands of individual genome sequences and are integrated into the Ensembl infrastructure for genome annotation and visualisation. The database and software system is easily expanded to integrate both public and non-public data sources in the context of an Ensembl software installation and is already being used outside of the Ensembl project in a number of database and application environments.

**Conclusions:**

Ensembl's powerful, flexible and open source infrastructure for the management of variation, genotyping and resequencing data is freely available at http://www.ensembl.org.

## Background

The advances in sequencing technologies over the last decade have transformed biology into an information rich science and created the field of bioinformatics. In parallel to this, storage requirements have grown from storing a human genome reference sequence [1,2], to handling the increasing volumes of variation data. Earlier projects used dense genotyping technologies on individuals (notably using the Perlegen [3] and HapMap [4] samples). More recently, large-scale resequencing of whole genomes and selected functional regions is taking place in the 1000 Genomes Project for human [5] and 1001 Genomes Project for Arabidopsis [6]. Genome-wide association studies, such as that performed by the Wellcome Trust Case Control Consortium (WTCCC) [7], are using the recent generation of genotyping chips and creating data sets on a new scale.

There is a demand for databases designed to manage the growing amount of variation information. An infrastructure is necessary that facilitates data analysis and integration with existing genome annotation. Storing dense variation data for many individuals in any naive manner requires a large amount of disk space, a requirement that is only likely to increase in coming years.

### Public Resources of Variation Data

In parallel with advances in data production technology, the bioinformatics infrastructure required to manage, organise and analyse the data has also evolved. A number of databases were originally designed to capture variation data and organise it with respect to the genome assembly. Ultimately the effort and expense required to maintain and continually update these resources over time were not available, and many of the previous public databases of variation data, such as those provided by The SNP Consortium [8] and HGVbase [9], have either changed their focus or stopped active development. Indeed, the management of large-scale variation data is currently limited to only a few projects including Ensembl [10], the UCSC Genome Browser [11] and dbSNP [12]. In addition, there are numerous highly curated and special purpose databases, including locus-specific databases [13] and those dedicated to specific applications, such as SeattleSNPs which focuses on associations between SNPs and inflammatory response pathways [14].

The most comprehensive resource is dbSNP, which serves as both a publication and permanent archive for variation data. Access to the data is provided visually, via a web interface, and also for bulk data download from an FTP site, allowing users to create a local copy of the data. The physical size of dbSNP (build 130 for human is approximately 230 GB) and its complex schema structure means that creating a local copy is generally a job for an experienced programmer with significant computational resources [15].

The UCSC Genome Browser provides access to the dbSNP data in the form of an annotation track. The data is made available after each dbSNP build is completed and is provided both visually through the browser interface and by data download. Variation data mining is supported at UCSC through the use of tools such as the table browser [16].

### Ensembl

Ensembl is a comprehensive genome information system that provides variation data resources alongside many other interconnected components, including the Ensembl Genome Browser at http://www.ensembl.org. Ensembl is updated approximately five times each year with new genome assemblies and additional data as it becomes available. In release number 57, Ensembl fully supports over 50 genomes, of which 14 currently have variation data associated with them. In order to integrate variation data into the existing Ensembl resources, a dedicated variation database and API is required to support the web site and other bioinformatics tools [17].

**Figure 1:**
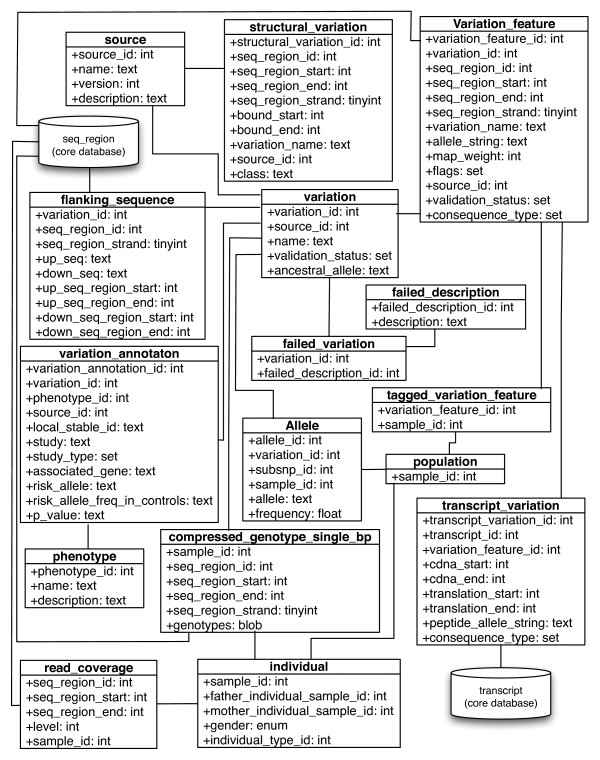
**Simplified Database Schema**. A simplified version of the Ensembl variation database schema. Sequence variants are stored in the variation table shown in the top centre of the figure. A sequence variant is defined as having a source, flanking sequence and at least one allele. Structural variants have their own table. Connections to the Ensembl core database for sequencing information and genome annotations are highlighted. The full database schema is provided as a supplemental file and available at http://www.ensembl.org/info/docs/api/variation/variation_schema.html.

This report presents a detailed description of the Ensembl variation database and Application Programming Interface (API). As with the other Ensembl databases, the variation database is based on an open source MySQL database [18] infrastructure. The variation database schema is presented in summary in Figure 1 and the full schema is included as Additional file 1: Supplemental Figure S1. It is designed explicitly to deal with both large-scale, dense genotyping data and resequencing data covering thousands of individual genome sequences. The database provides all of the data visible on the Ensembl web site, as well as the supporting data that is only available through the Ensembl API. The API is one of the distinguishing features of Ensembl and provides a programmatic interface to all Ensembl data across all supported organisms. As a result, a script based on a given version of the API will work with all databases corresponding to that version and there is no need for species-specific programming. There is also a data-mining tool, BioMart [19], which is tightly integrated with the data resources.

## Implementation

### The Variation API

The variation API is designed for optimal interaction with the database and provides a powerful and comprehensive interface to the variation data. It is written in object-oriented Perl [20] and follows the same general conventions as the wider Ensembl API [21].

**Figure 2:**
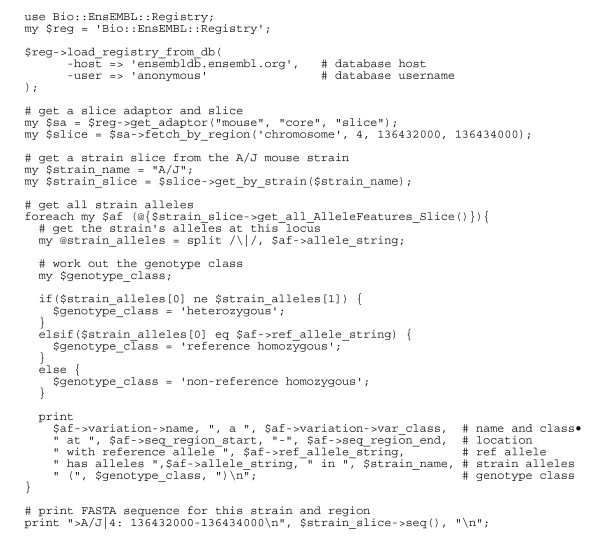
**Using the API to access variation and sequence**. This example describes how to use the registry and the API to connect to an Ensembl database and access and analyse variation from a specific region of the genome in a given individual sequence.

Figure 2 gives an example of how to use the Ensembl API. Like the other components of the Ensembl API, the variation API leverages the Ensembl Registry module to connect to the requested databases and import the relevant Perl modules. This feature allows the user, in only a few lines of code, to connect to either the public Ensembl databases at ensembldb.ensembl.org or to any custom databases held on the user's local file system. Once connected, the Registry object can be used to create a series of object adaptors. These object adaptors act as "factories" for generating objects that represent entities in the underlying database; for example, a variation adaptor object can be used to generate variation objects representing variants stored in the database: similarly an allele adaptor generates allele objects.

Multiple retrieval methods are provided in each of the object adaptors, allowing the user to create, for example, single variation objects given a specific identifier, or sets of objects such as a list of all variation feature objects mapped within a particular gene. All object types and methods are fully documented in online POD documentation, which is also available in HTML format via the Ensembl website http://www.ensembl.org/info/docs/Pdoc/index.html, along with a series of tutorials introducing the user to the API http://www.ensembl.org/info/docs/api/index.html.

The example code in Figure 2 shows how the Ensembl API can be used to retrieve sequence variation from a specific mouse strain and then, for each locus, print how the strain compares to the reference sequence. First, the API creates a slice (a contiguous region of the genome representing the chromosome) for the genomic region of interest. Then, the get_all_AlleleFeatures_Slice method is called to retrieve the alleles for this genomic slice. The example code classifies the genotype at each locus using a general algorithm, which could be used for both homozygous inbred genomes such as laboratory mouse strains or outbred heterozygous individuals. Finally, the example code outputs the resulting variants and sequence for the non-reference individual.

The DB Connection adaptor also supports the attachment of multiple variation databases supporting, for example, seamless integration of publicly available databases at ensembldb.ensembl.org and internal data which may be subject to access restrictions due to patient confidentially or other reasons. Full details of the Ensembl API conventions are available at http://www.ensembl.org/info/docs/api/index.html. The API is used by the Ensembl website http://www.ensembl.org and can also be used for custom analysis, on any combination of the public databases or the user's own data.

## Storage and Retrieval of Variation Data

### Database requirements and design considerations

Ensembl stores a variety of different data types including variants on the genome from external sources such as dbSNP and those resulting from Ensembl's internal SNP calling pipeline [17]. Other data result from resequencing projects or dense genotyping projects that are based on Affymetrix, Illumina and other high-throughput genotyping platforms. In addition, the database provides the predicted consequences of each variant in the context of the evidence-based Ensembl gene annotations and a representation of resequencing information that is compact and supports a visual presentation. The way the different data types are stored in the variation database is discussed below while a more in-depth explanation of the data itself can be found in Chen et al [17].

### Variation data

In Ensembl, a sequence variant (SNP or in-del) is defined by its upstream and downstream flanking sequences along with at least one variant allele. The flanking sequence of each variant is aligned to one or more positions in the reference genome. To accurately reflect this relationship there exists in the schema two distinct table sets (Figure 1): the variation, flanking sequence and allele tables store the most basic information for each variant in the database independently of its position on an assembly, while the variation feature table contains mappings for each variant to the reference sequence. This division of data means that only the variation feature table needs to be updated when there is a new assembly released and the variation table remains consistent regardless of the assembly.

Mappings for each variant are imported directly from dbSNP or are generated by aligning the composite 5' flanking sequence, reference allele and 3' flanking sequence to the reference sequence using the ssaha2 alignment software [22]. After import or mapping, the size of the flanking sequence table can be greatly reduced by replacing raw sequences with a set of coordinates when the sequence exactly matches the reference sequence at its mapped location. As discussed below, the API seamlessly restores the flanking sequence not directly stored in the database with the appropriate sequence drawn from the associated Ensembl core database.

When a variant maps at least once, but no more than *N *times, these mappings are stored in the variation feature table (*N *= 3 is currently used for all supported species). Variants that fail to map to the genome, or map more than N times, are recorded in the failed variation table and no mappings will be stored in the variation feature table for the given variant. Further quality control criteria are also applied: variants with alleles that do not match the reference sequence at the location of mapping are also moved to the failed variation table (see Chen et al [17] for details on how and why variants are assigned entries in the failed variation table). A 'white list' of variants, such as those with clinical significance, will not be moved to the failed variation table even if they meet one of the above criteria. All supplementary data pertaining to variants in the failed variation table, including alleles and genotypes, are deleted from the database before release.

Ensembl also stores structural variants and copy number data. The location of these features is harder to define due to the uncertainly of their boundaries. For this reason, they are stored in a separate structural variation table which allows for start and end positions to be defined as a range between a minimum and maximum value.

### Individuals and population genotypes: Genotype compression

Large-scale and dense genotype data, such as that produced by the HapMap Project, requires significant disk space to store in a naïve database implementation or as flat-file data [23]. In the initial implementation, Ensembl kept all genotype data in a table designed to store single base pair genotypes using an approach that dedicated one row per genotype in the table. Based on the more than 1.5 billion human genotypes available in Ensembl release 57 (March 2010), this single table would currently be more than 30 Gb in size. Moreover, accessing the table to provide data for the Ensembl web displays [17] would require impractical database lookup times.

To address this problem, a simple and efficient compression algorithm was created for storing an encoded representation of the genotype data. This compact structure, discussed below, reduces the size of the table to only 12 million entries, which require approximately 5 Gb of disk space.

Each row in the compressed table stores genotypes from one individual in one fixed-size region of the genome (arbitrarily defined as 100 Kb). A binary field in the row stores a compressed string (using Perl's pack method) consisting of a repeating triplet of elements: a distance in base pairs from the previous genotype followed by a pair of alleles. Using distances rather than absolute chromosomal positions minimises the number of bytes required to store these integers. Along with fields describing the position of the given region, methods in the API can decode the compressed string into a set of genotypes at any position in the genome.

For example, a given row may have a start position of 1000, indicating the chromosomal position of the first genotype in this row. The unpacked genotypes field then may contain the following elements:

The first genotype has a position of

and alleles A and G. The second genotype has a position of

and alleles C and C, the third genotype similarly has a position of 1055 and alleles G and T, the fourth position 1375 and alleles A and A, and so on.

In practice, since the first genotype in the set will always be at the chromosomal position specified in the row's position field, the 0 shown above is not explicitly stored, but is added by the API as part of the decoding process.

In this way the database is optimised for the efficient access of all of the data in a particular genomic region, a decision made in part to enable optimal real-time display of the data in the Ensembl genome browser.

Genotypes for other types of variants, including insertions, deletions, repeat features and larger structural variants are stored in a separate, uncompressed multiple base pair genotype table.

### Population Level Data

Population-level data is also stored in the schema. Specifically, the allele table stores observed population allele frequencies, while the population genotype table stores observed genotype frequencies. These frequencies are imported from dbSNP, and hence include reference populations from sources such as the HapMap Project and those genotyped by Perlegen.

### Real-Time calculation of linkage disequilibrium

**Figure 3:**
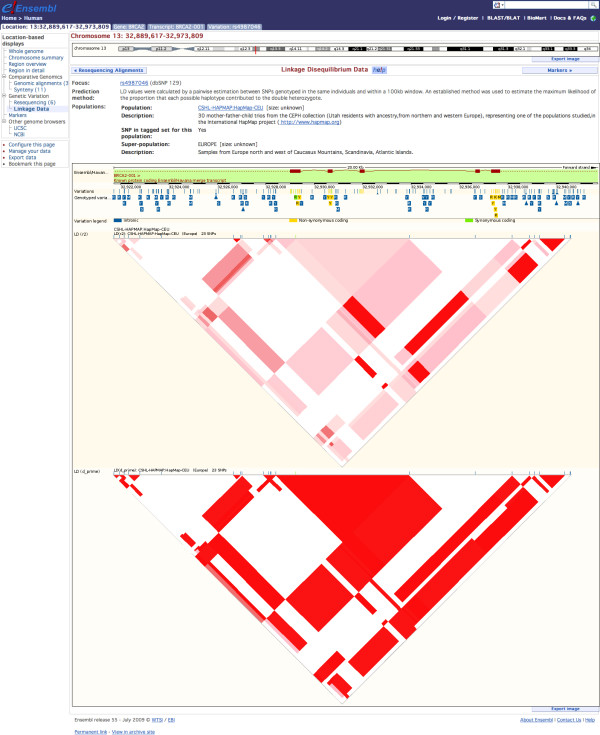
**Linkage Disequilibrium Plot**. The pattern of linkage disequilibrium (LD) in the HapMap CEU population for a 20 kb region of the BRCA2 gene. The displayed values for r^2 ^and D' are calculated on the fly by the variation API, which calls a dedicated C function after the necessary data is extracted from the database.

Linkage disequilibrium (LD) data provided through the Ensembl web interface is calculated "on-the-fly" using a highly efficient C program. Calculating LD data in such a manner negates the need for a large and unwieldy table containing millions of pairwise r^2 ^and D' values. The program implements a version of the standard EM algorithm for the estimation of pairwise haplotype frequencies from unphased genotype data, providing a similar level of accuracy to that seen in the popular Haploview program [24]. LD data can be produced in this way via the API, or be visualised in the classic 'inverted triangle' display via the web interface (Figure 3).

### Resequencing data

As the cost of DNA sequencing has reduced dramatically over the last decade, an increasing number of resequencing studies are being performed, generating many new variants [25]. Although resequencing data in the form of sequencing reads is normally stored in the European Nucleotide Archive (ENA) http://www.ebi.ac.uk/embl/ or Genbank http://www.ncbi.nlm.nih.gov/Traces/, optimal representation of these data should take into account that the vast majority of the sequence will be the same as the reference assembly. Thus the database stores only two components of the resequencing data, which allow the recreation of essentially all of the information: (1) alignment positions of the sequence reads, represented as coordinates with read coverage levels, and (2) differences between the alignments and the reference genome assembly, stored as variations. These differences may be single nucleotide polymorphisms, insertions and deletions or larger structural variations.

### Phenotypic Information

The schema also allows for the storage of phenotypic information associated with particular variants, along with any relevant association data such as risk alleles and p-values, in the variation annotation table. Multiple annotations may be linked with an individual variation object in the database for such situations as when alleles may have different disease associations. These phenotype data are fully indexed and searchable through the Ensembl web interface.

### Haplotypic and pseudo-autosomal regions

The Ensembl core database schema includes an explicit representation of haplotypic regions that appear, for example, in the MHC region of human chromosome 6. This data structure allows for these regions (and the pseudoautosomal regions of the X chromosome) to be stored and managed as efficiently as possible. The variation database supports this data structure by allowing multiple haplotypic variation feature objects to correspond to one variation object, but correctly considers that each of the variation objects only map to a single place in the reference genome. Importantly, this data structure supports the appropriate variations on the various haplotypes.

### Consequences of variants

The tight integration of the Ensembl variation database and API with the other components of the Ensembl architecture allows the derivation of novel and biologically relevant data. Methods in the API allow the prediction of consequence types of variants on the transcripts to which they map by assessing the change that each variant allele causes in the reference sequence. These consequence types include, but are not limited to: non-synonymous amino acid changes in protein coding transcripts; stop codon gain or loss; frame shifts caused by insertions or deletions; variants located within splice sites or regulatory regions. From a variant's position with respect to a transcript, a consequence type is calculated and stored in the transcript variation table. In addition, Ensembl is unique in providing an API method to predict consequence types for novel variant positions, given a genomic location and an allele.

## Results and Discussion

The Ensembl model consists of one variation database for each supported species with the associated genome sequence data and genome annotations, such as protein coding genes, available in the Ensembl core databases (see Figure 1). The variation databases vary in size depending on the amount of data available for a given species and range, for example in release 57, from a size of 34 Gb for human, to 324 Mb for zebrafish.

Ensembl is designed to be used for data access in a variety of methods, serving the diverse requirements of the scientific community. The web interface caters to those who wish to view variation data in the context of the other genomic annotation offered by Ensembl, and offers unique representations and visualisations of the data. For those wishing to extract large volumes of data, BioMart can be used to filter and retrieve such data through a highly configurable interface. The variation API can be used in conjunction with the other Ensembl APIs to make queries across all the databases, offering programmatic access to the full range of data stored in Ensembl's public databases. The large Ensembl codebase can be incorporated into pipelines and custom software, as well as providing a comprehensive and expandable interface to a user's own data.

The variation database schema and API are regularly updated to include new functionality and refinements of existing methods. These software updates are released in sync with the rest of Ensembl and in conjunction with updates to the data content of variation databases. Thus each release consists of new data, changes associated with new genome assemblies and annotation and the corresponding changes to the API. Taken together these resources provide a comprehensive and unique solution for the management and analysis of variation data.

The database and API described here share some functionality with the tools available for other genome browsers. For example, the UCSC genome browser stores variation data in tables that represent locations of variation, genotyped SNPs, etc [26]. These tables can be visualised as tracks on the UCSC Genome Browser [11] or used by the UCSC Table Browser to create more complex queries across multiple data tables available at UCSC [16]. However, the full flexibility of analysis enabled by the variation API is unique to Ensembl as are the federated queries that are available through the Ensembl variation BioMart [19]. Moreover, the variation objects described here can be implemented with an identical interface by other projects. In this way the variation objects can serve as "common currency" for a consistent analysis across data available in a number of different physical locations each stored in databases with different schema and potentially different database platforms.

The variation database and API are currently deployed in a number of projects beyond Ensembl. For example, they provide a portion of the variation data infrastructure for the 1000 Genomes Project [27] and the Gramene Project [28].

### Future

As the cost of sequencing technologies decreases and the sequencing of individual genomes from the 1000 Genomes Project and other projects becomes more commonplace, it is likely that further development will be required in order to maintain optimal data storage efficiency while also being able to retrieve the data in a timely fashion. In addition, there will be additional developments in the API for new data queries such as retrieving summary information for variants across a number of individuals from the same population or to retrieve the sequence of a particular individual, breed or strain.

Although Ensembl focuses on chordate species, the recently launched Ensembl Genomes project at the EBI will extend Ensembl technology to the analysis of a wider variety of genomes [29]. These requirements will necessitate extension of the capabilities of the variation database and API to support specific characteristics of non-diploid genomes such as those found in plants and yeast.

## Conclusions

Ensembl's tools for variation and resequencing data are designed to solve the large-scale data analysis, storage and visualisation challenges presented by current and next generation genotyping and sequencing platforms. The platform is extensively tested and heavily used as the software library and database interface for the Ensembl genome browser and other projects built with Ensembl technology.

## Availability and requirements

Project Name: Ensembl

Project homepage: http://www.ensembl.org.

Operating system: Platform independent

Programming language: The Ensembl API is written in Perl with supporting C functions as described above.

Other requirements: The Ensembl API requires BioPerl 1.2.3. Additional requirements apply to users wanting to install a full Ensembl mirror site as described at http://www.ensembl.org/info/docs/webcode/index.html.

License: All of the code described in this article is freely available under the terms of the Ensembl software license found at http://www.ensembl.org/info/about/code_licence.html

## Additional Information and On-line Tutorials

The database schema is described here in http://cvs.sanger.ac.uk/cgi-bin/viewcvs.cgi/ensembl-variation/schema/. http://www.ensembl.org/info/docs/api/variation/index.htmlhttp://www.ensembl.org/info/docs/api/variation/variation_tutorial.html.

## Authors' contributions

The database schema was designed by AS, EB, DR and WM with contributions from YC, PF and FC. The API was written by DR with contributions from AS, WM and YC. YC, WM and DR have built the variation databases available at Ensembl. PF, WM and FC wrote the paper with contributions from DR and input from all of the other authors. All authors have read and approved the final manuscript.

## Supplementary Material

Additional file 1 **Supplemental Figure S1**: Full Variation Database Schema. Click here for file
