# Supplementary figures and images for: A database and API for variation, dense genotyping and resequencing data
Source: BMC Bioinformatics. 2010 May 11;11:238. doi: 10.1186/1471-2105-11-238 (PMC2882931; doi:10.1186/1471-2105-11-238)

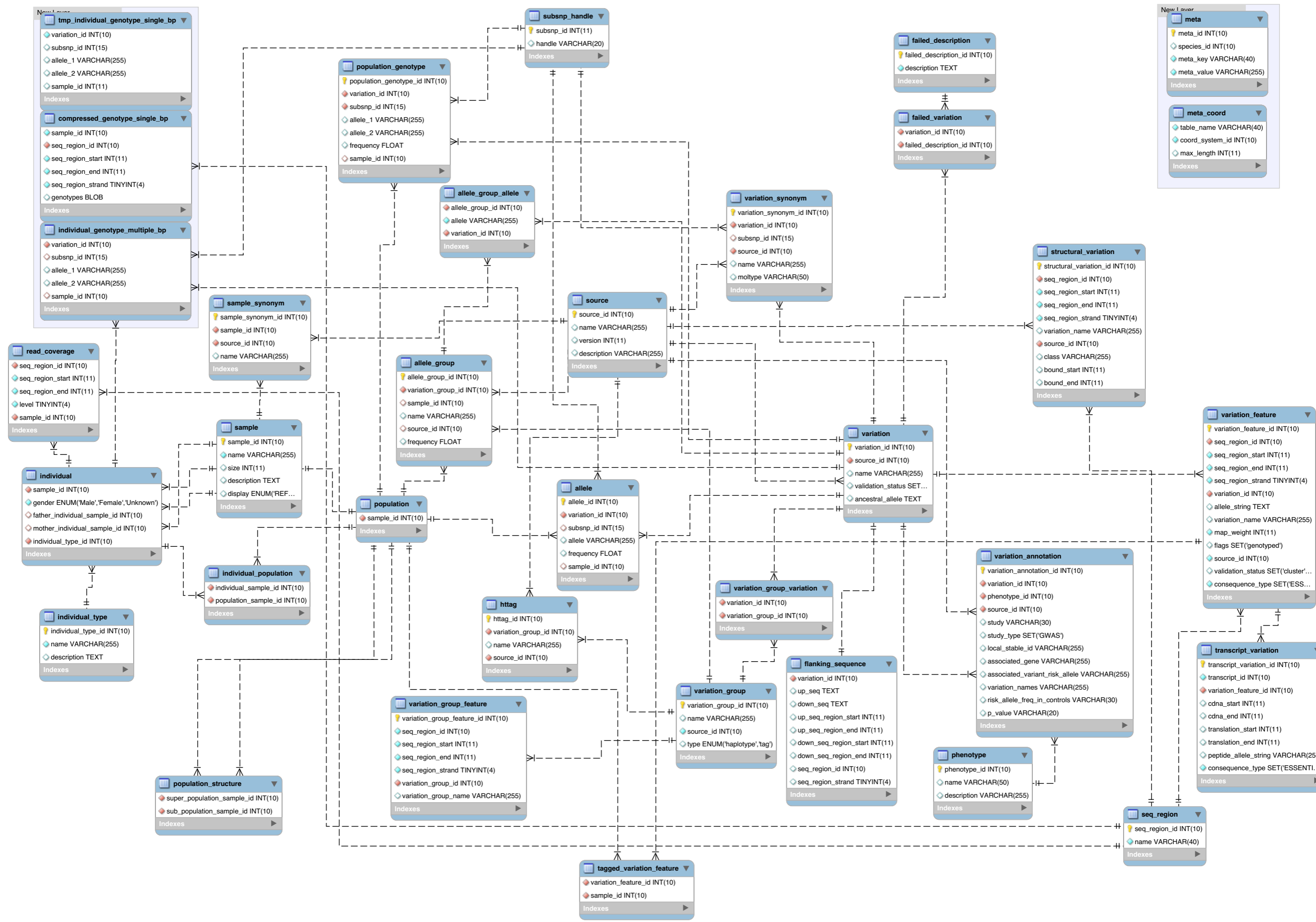

Supplement: Additional file 1 — Supplemental Figure S1: Full Variation Database Schema. [file 1471-2105-11-238-S1.PDF]
